# Supplementary material for: High-resolution metagenome assembly for modern long reads with myloasm
Source: bioRxiv. 2025 Sep 6:2025.09.05.674543. Preprint. [Version 1] doi: 10.1101/2025.09.05.674543 (PMC12424758; doi:10.1101/2025.09.05.674543)
Supplement: 1 [file NIHPP2025.09.05.674543V1-supplement-1.pdf]

bioRxiv preprint doi: https://doi.org/10.1101/2025.09.05.674543; this version posted September 6, 2025. The copyright holder for this preprint (which was not certified by peer review) is the author/funder, who has granted bioRxiv a license to display the preprint in perpetuity. It is made available under aCC-BY 4.0 International license.



## Supplementary Materials

### Proof of Theorem 1

**Theorem 1.** Let $M_n$ be the number of SNPmers with a mismatched middle base for a random string of length $X$ of length $n$. Let $O_n$ be the analogous random variable that counts the number of matching open syncmers. Set

$$R_n = \frac{M_n}{O_n \cdot c}.$$

Under an idealized model of $X$ where every k-mer and s-mer is unique, as $n \to \infty$,

$$\frac{R_n}{1 + R_n} \xrightarrow{p} \theta.$$

That is, the left hand side converges to the right hand side in probability.

*Proof.* Under an idealized random string model with k-mers and s-mers represented as uniformly random hashes, Spouge et al. [83] proved a central limit theorem (CLT) for "downsampled" (analogous to sequencing errors in our case) open syncmer matching:

$$\frac{O_n}{\sqrt{n}} \to N(\frac{\sqrt{n}}{c} \cdot (1-\theta)^k (1-\epsilon)^k, \sigma_M)$$

for some $\sigma_M$.

Our strategy of proving Theorem 1 is as follows. Firstly, the above CLT implies that the law of large numbers as a corollary:

$$O_n/n \to \frac{1}{c} \cdot (1-\theta)^k \cdot (1-\epsilon)^k$$

in probability. We will only need the law of large numbers, not the CLT.

We will show that

$$M_n/n \to \theta(1-\theta)^{k-1}(1-\epsilon)^k \quad (10)$$

in probability by proving a CLT theorem for the SNPmers. Assuming this convergence holds,

$$R_n = \frac{M_n}{n}/(c \cdot \frac{O_n}{n})$$

converges to

$$R_n \to \theta/(1-\theta)$$

in probability by Slutsky's theorem. Then the continuous mapping theorem with the function $x \mapsto x/(1+x)$ shows that $R_n/(1+R_n)$ converges to $\theta$, and we are done. Thus, our job is to prove Equation 10.

To prove Equation 10, we follow the same framework as Spouge et al. [83] and use a dependent central limit theorem of Hoeffding and Robbings [108] to prove that $M_n/\sqrt{n}$ approaches a Gaussian.

**Supplementary Lemma 1 (Hoeffding-Robbins CLT).** *Given a sequence of random variables $Z_1, ..., Z_n$, this sequence is k-dependent if $r - s > k$ implies that $Z_{i+r}$ and $Z_{i-s}$ are independent for any $i$. Assume that $\mathbb{E}[Z_i] = 0$ and $\mathbb{E}[|Z_i|^3] < \infty$. Define*

$$A_i = \mathbb{E}[Z_{i+k}^2] + 2\sum_{j=1}^{k} \mathbb{E}[Z_{i+k} Z_{i+k-j}].$$

*Assume that $\lim_{n\to\infty} \frac{\sum_{i=1}^{n} A_i}{n} = A < \infty$ exists. Then, $\frac{1}{\sqrt{n}}(Z_1 + ... + Z_n)$ has a limiting normal distribution with mean 0 and variance $A$.*

bioRxiv preprint doi: https://doi.org/10.1101/2025.09.05.674543; this version posted September 6, 2025. The copyright holder for this preprint (which was not certified by peer review) is the author/funder, who has granted bioRxiv a license to display the preprint in perpetuity. It is made available under aCC-BY 4.0 International license.



Along the random string $X$, our idealized SNPmers mismatches are $k$-dependent; this is because outside of this k-mer, the bases do not overlap and the letters are independent. Let $Z_i = z_i - p$ where $p = \theta(1-\theta)^{k-1}(1-\epsilon)^k$ and $z_i$ is an indicator random variable that takes 1 if a SNPmer mismatch is found at position $i$. Clearly $\mathbb{E}[Z_i] = 0$ by our previous discussion of $\mathbb{E}[z_i]$. $\mathbb{E}[Z_i^3] < \infty$ also holds because $Z_i^3$ is an indicator random variable. We just need to show that $\sum_{i=1}^{n} A_i/n < C$ for some $C$ and all $n$. The following inequality is sufficient:

$$\mathbb{E}[Z_i Z_j] = \mathbb{E}[z_i z_j - p(z_i + z_j) + p^2] \leq \mathbb{E}[z_i] - 2p^2 + p^2 = p - p^2.$$

This holds because $z_i z_j \leq z_i$ for all $i, j$ (we are multiplying 0-1 indicators). Thus,

$$\sum_{i=1}^{n} A_i/n \leq (2k+1)(p+p^2)$$

for all $n$, showing that $M_n/\sqrt{n} \to N(p\sqrt{n}, A)$ for some constant $A$. By CLT implying law of large numbers, this also proves Equation 10. This implication finishes the proof by the above argument.

| **Dataset (ONT R10.4)** | **Accession** | **Dataset (HiFi)** | **Accession** |
|---|---|---|---|
| Oral 1 (Kiguchi et al.) | DRR582205 | Hot Spring (Kato et al.) | DRR290133 |
| Oral 2 (Kiguchi et al.) | DRR582179 | Anaerobic Digester (Benoit et al.) | ERR10905741 |
| Gut 1 (Minich et al.) | SRR29980972 | Chicken Gut (Zhang et al.) | SRR19683891 |
| Gut 2 (Minich et al.) | SRR29980959 | Human Gut 4 (Gehrig et al.) | SRR15489018 |
| Gut 3 (Minich et al.) | SRR29980980 | Seawater 1 (Priest et al.) | ERR4920901 |
| Microflora (Sereika et al.) | ERR11523665 | Seawater 2 (Sidhu et al.) | ERR9769281 |

Supplementary Table 1: Datasets for real ONT and HiFi datasets.

bioRxiv preprint doi: https://doi.org/10.1101/2025.09.05.674543; this version posted September 6, 2025. The copyright holder for this preprint (which was not certified by peer review) is the author/funder, who has granted bioRxiv a license to display the preprint in perpetuity. It is made available under aCC-BY 4.0 International license.



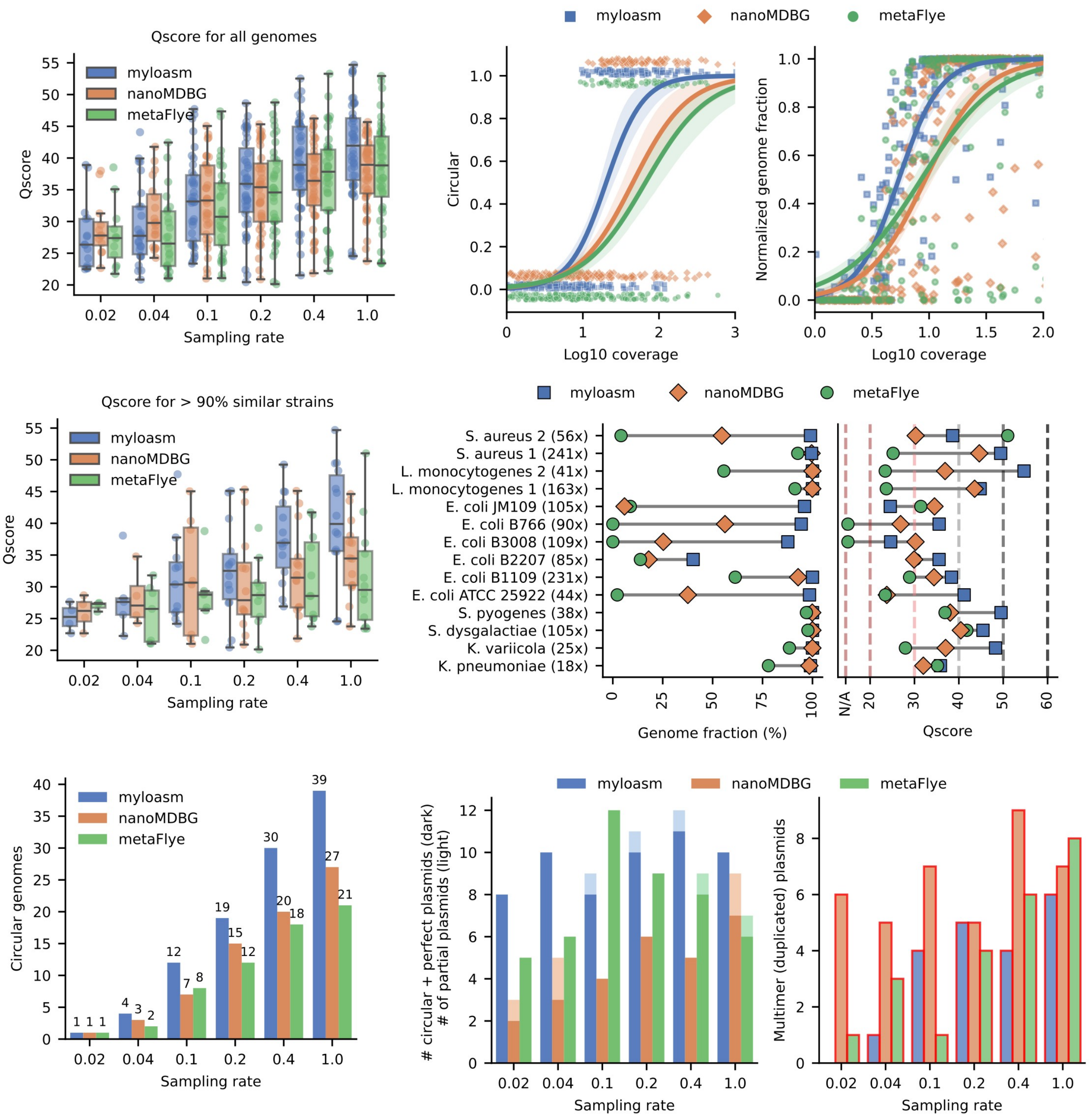


Supplementary Figure 1: Results for the R10.4 ONT community but with hac (high accuracy) basecalling instead of sup (super high accuracy) basecalling. All subfigures are generated in the same way as in **Fig. 2**.

bioRxiv preprint doi: https://doi.org/10.1101/2025.09.05.674543; this version posted September 6, 2025. The copyright holder for this preprint (which was not certified by peer review) is the author/funder, who has granted bioRxiv a license to display the preprint in perpetuity. It is made available under aCC-BY 4.0 International license.



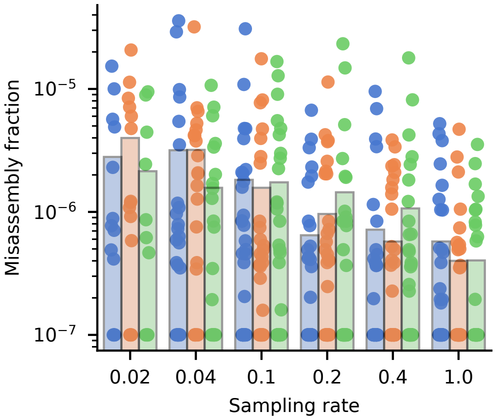


Supplementary Figure 2: Misassembly fraction (# of misassemblies per base pair) over all genomes with Qscore > 20 for the mock metagenome (sup basecalling). Genomes without misassemblies were set to $10^{-7}$ misassembly fraction. The bar height represents the mean value.

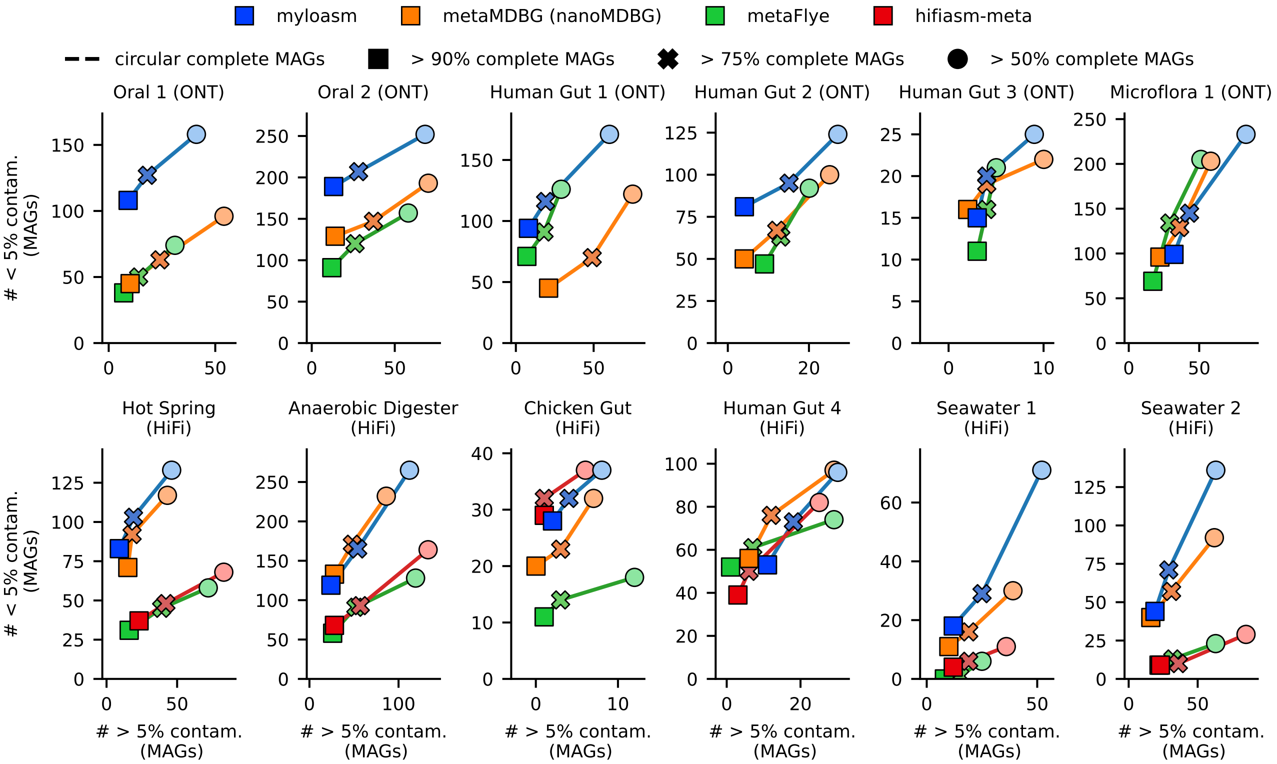


Supplementary Figure 3: Analogous contamination and completeness results to **Fig. 4A** but for *MAGs*, not contigs.

bioRxiv preprint doi: https://doi.org/10.1101/2025.09.05.674543; this version posted September 6, 2025. The copyright holder for this preprint (which was not certified by peer review) is the author/funder, who has granted bioRxiv a license to display the preprint in perpetuity. It is made available under aCC-BY 4.0 International license.



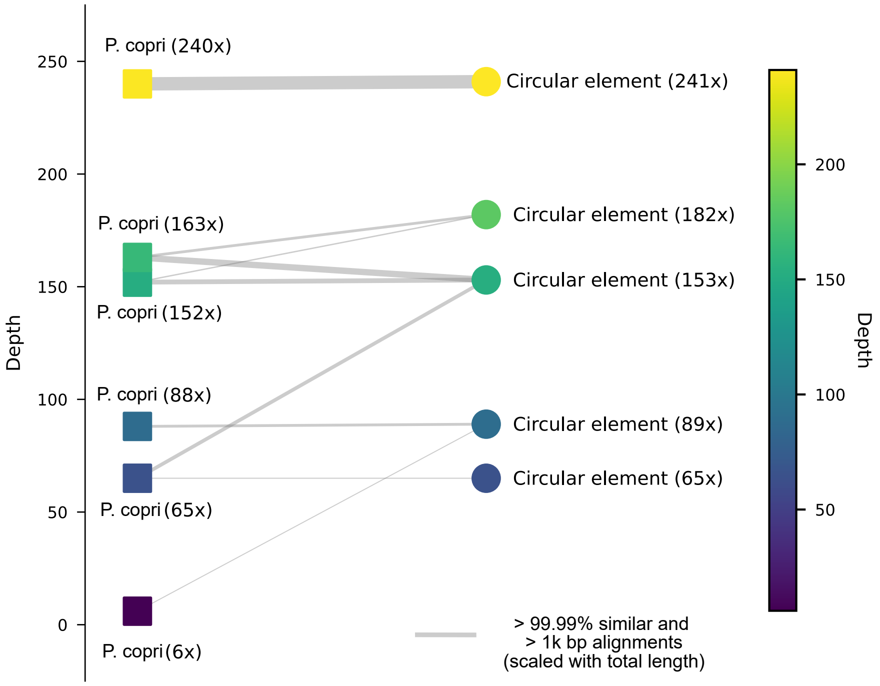


Supplementary Figure 4: Linking *P. copri* genomes to circular genomic elements of length $100 - 150$ kbp. Coverage was taken from myloasm's estimated coverage (denoted as DP3 in the output). We confirmed the similarity of the 241x coverage circular contig against known extrachromosal elements of *P. copri* from Blanco-Miguez et al. [61]. We then found four more circular elements within the assembly with $> 15\%$ aligned fraction to the 241x element. Lines are drawn for matches of $> 99.99\%$ similarity as found by minimap2 between all circular elements and *P. copri* genomes, with thickness scaled linearly with total matching bases.

bioRxiv preprint doi: https://doi.org/10.1101/2025.09.05.674543; this version posted September 6, 2025. The copyright holder for this preprint (which was not certified by peer review) is the author/funder, who has granted bioRxiv a license to display the preprint in perpetuity. It is made available under aCC-BY 4.0 International license.



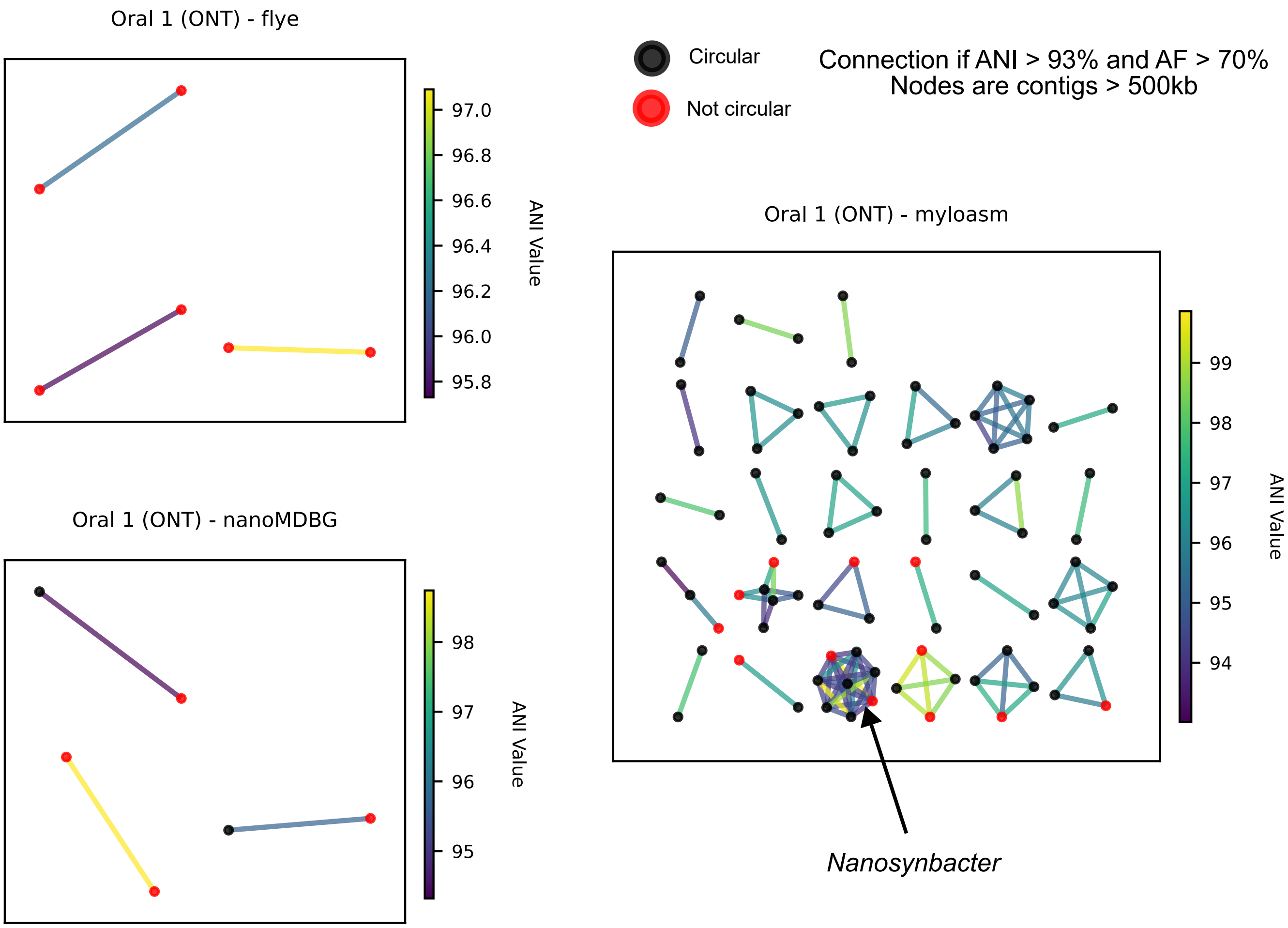


Supplementary Figure 5: Contigs of length 500kbp in the Oral 1 dataset with at least one other contig with $>93\%$ average nucleotide identity (ANI) and $>70\%$ aligned fraction. Black dots are circularized genomes; red dots are non-circularized. Edges are drawn between genomes with $>93\%$ ANI and $>70\%$ aligned fraction and are colored by ANI.

bioRxiv preprint doi: https://doi.org/10.1101/2025.09.05.674543; this version posted September 6, 2025. The copyright holder for this preprint (which was not certified by peer review) is the author/funder, who has granted bioRxiv a license to display the preprint in perpetuity. It is made available under aCC-BY 4.0 International license.



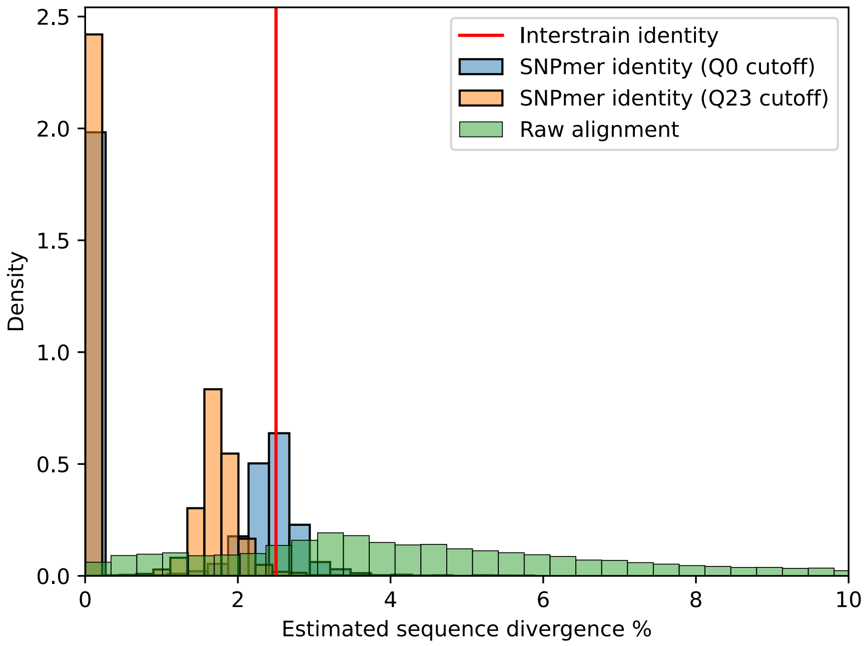


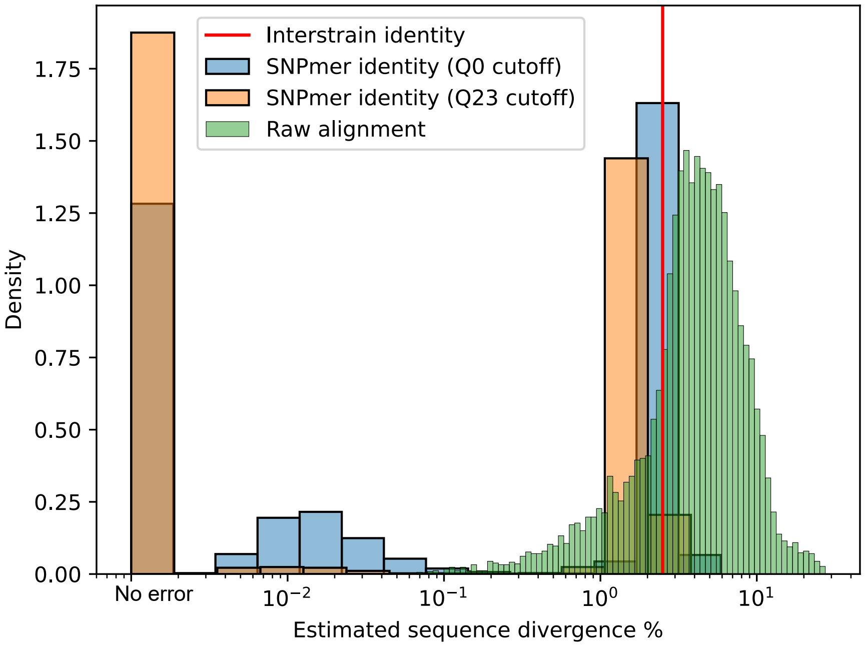


Supplementary Figure 6: The SNPmer divergence ($\hat{\theta}$) estimator (Equation 5) versus raw sequence identity for simulated reads from two *E. coli* genomes with 97.5% nucleotide similarity. Top: linear x-scale. Bottom: log x-scale. The second *E. coli* genome was generated by simulating substitutions with probability 0.025. Reads for both genomes were simulated with badread and default parameters except with mean accuracy 98%; the two read sets were then concatenated. Raw sequence identity was calculated by all-to-all read alignments of the strain-mixed reads with minimap2. We show two different values of $\hat{\theta}$: one after removing SNPmers with middle base quality $< 23$, and one with no base quality restrictions. Thresholding base qualities leads to a biased estimate of sequence divergence, but it also removes lots of low divergence overlaps ($10^{-2}\% - 10^{-1}\%$) that are from substitution sequencing errors (bottom).

bioRxiv preprint doi: https://doi.org/10.1101/2025.09.05.674543; this version posted September 6, 2025. The copyright holder for this preprint (which was not certified by peer review) is the author/funder, who has granted bioRxiv a license to display the preprint in perpetuity. It is made available under aCC-BY 4.0 International license.



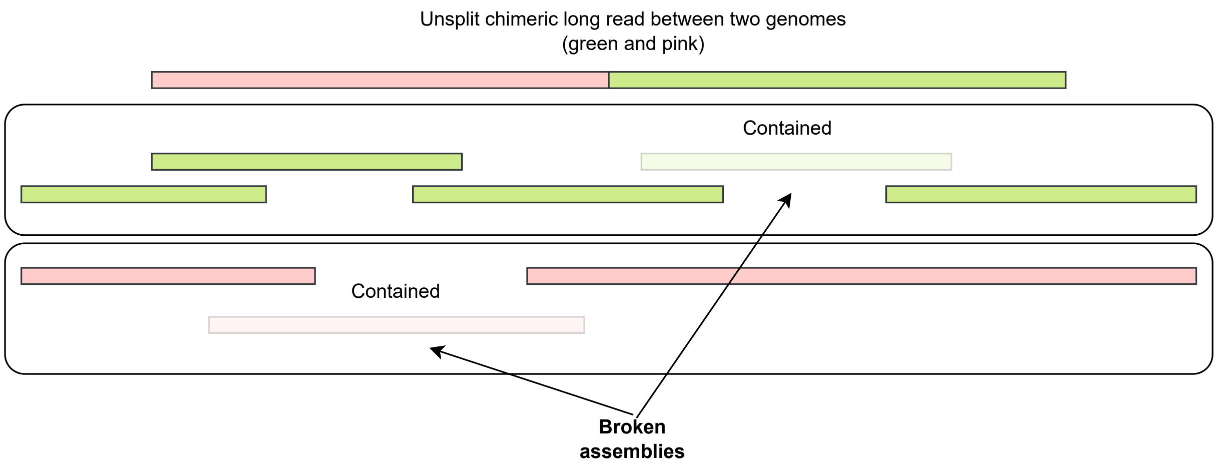


Supplementary Figure 7: Illustration of how large structural errors within reads can cause misassemblies or fragmented assemblies. If a long chimeric read (top) is not split or removed, this read will remove smaller contained reads. The removal of the contained reads leads to broken assemblies, or the chimeric read may lead to a chimeric assembly.

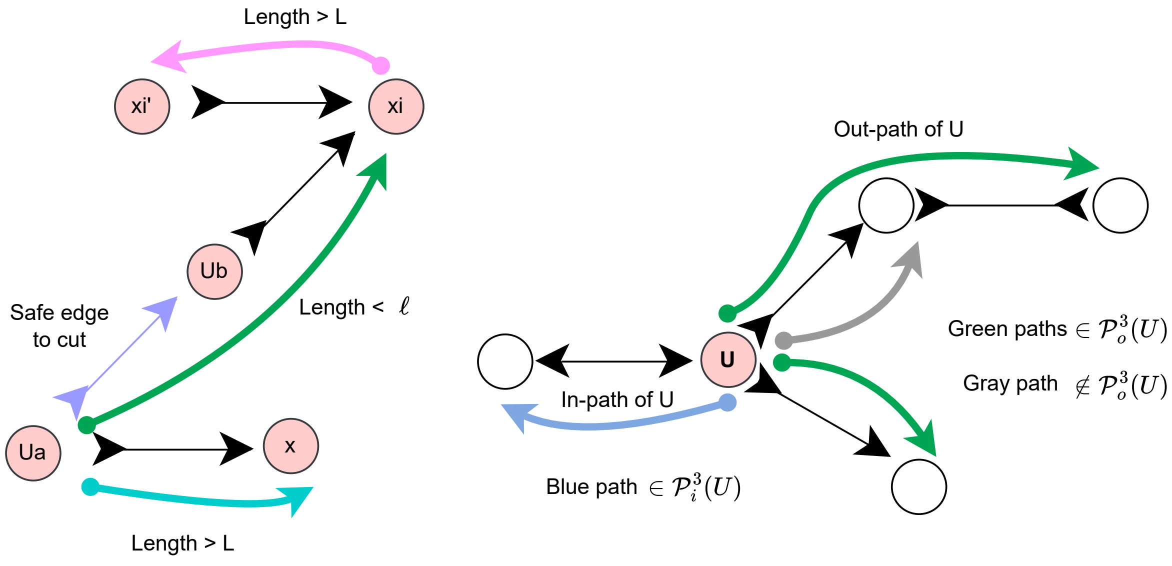


Supplementary Figure 8: Illustration of key concepts in the bidirected graph representation of read overlaps. Edges in bidirected graphs have two independent directions—one direction for each node. **Left**: illustrating the definition of safety (Definition 10). The purple edge is safe to cut if there exists (1) a long path out of $U_a$ (teal path) and (2) a long path (pink) that is "opposite" to a small path (green) out of $U_a$. **Right:** illustrating Definition 14. In-paths and out-paths are shown. The gray path is not "maximal" for $\mathcal{P}_o^3(U)$ because it can be extended to a path of length 3. Thus, the gray path has no associated probability under our model.

bioRxiv preprint doi: https://doi.org/10.1101/2025.09.05.674543; this version posted September 6, 2025. The copyright holder for this preprint (which was not certified by peer review) is the author/funder, who has granted bioRxiv a license to display the preprint in perpetuity. It is made available under aCC-BY 4.0 International license.



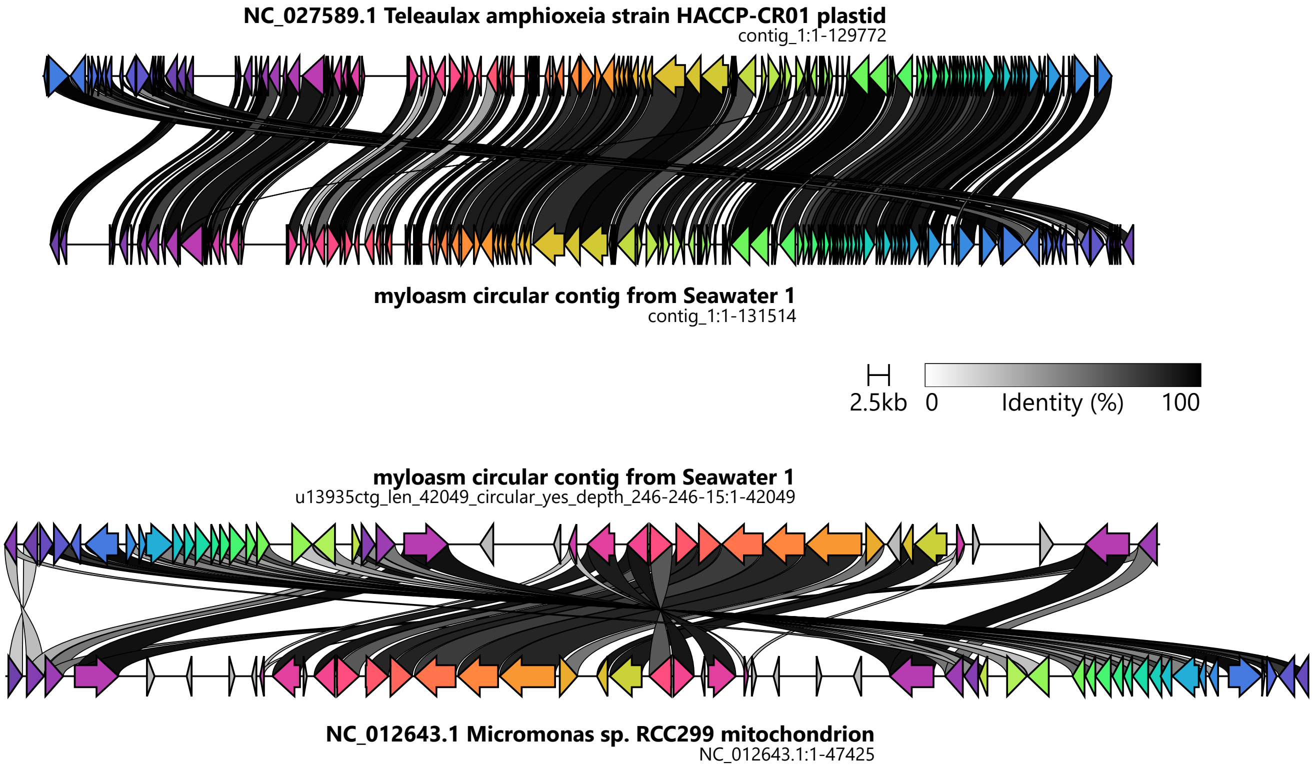


Supplementary Figure 9: Circular and complete plastid and mitochondrial genomes recovered from the Seawater 1 dataset. The reference genomes NC_027589.1 and NC_012643.1 were the top hits after blasting the genomes to the NCBI blastn standard database. The reference genomes had $80 - 85\%$ nucleotide similarity to myloasm's contigs. Plots were generated by clinker [105].
